# Pro-vaccination personal narratives in response to online hesitancy about the HPV vaccine: The challenge of tellability

**DOI:** 10.1177/09579265231181075

**Published:** 2023-09-08

**Authors:** Elena Semino, Tara Coltman-Patel, William Dance, Zsófia Demjén, Claire Hardaker

**Affiliations:** Lancaster University, UK; Lancaster University, UK; Lancaster University, UK; University College London, UK; Lancaster University, UK

**Keywords:** HPV, narrative, online forums, tellability, vaccination

## Abstract

Experimental studies have shown that narratives can be effective persuasive tools in addressing vaccine hesitancy, including regarding the vaccine against the human papillomavirus (HPV), which is transmitted via sexual contact and can cause cervical cancer. This paper presents an analysis of a thread from the online parenting forum Mumsnet Talk where an initially undecided Original Poster is persuaded to vaccinate their child against HPV by a respondent’s narrative of cervical cancer that they describe as difficult to share. This paper considers this particular narrative alongside all other narratives that precede the decision announced on the Mumsnet thread. It shows how producing pro-vaccination narratives about HPV involves challenges regarding ‘tellability’ – what makes the events in a narrative reportable or worth telling. We suggest that this has implications for the context-dependent nature of tellability, the role of parenting forums in vaccination-related discussions, and narrative-based communication about vaccinations more generally.

## Introduction

Storytelling is recognised as a central tool for making sense of and sharing personal and collective experiences. It can be used to perform a range of functions in different contexts, including moral teaching, community-building, escapism and persuasion. The role that narratives can play in healthcare and the experience of illness in particular is also well documented (Charon, 2008; Greenhalgh and Hurwitz, 1998). This includes the potential persuasive power of narratives in the context of vaccine hesitancy (Cawkwell and Oshinsky, 2016), defined by the World Health Organization as ‘the reluctance or refusal to vaccinate despite the availability of vaccines’ (World Health Organization (WHO), 2019).

In this paper we are concerned with narratives of personal experience told on a thread from the online parenting forum Mumsnet Talk in response to an Original Post in which a parent expresses indecision about vaccinating their daughter against the human papillomavirus (HPV). This thread is an unusually explicit, naturally-occurring example of the persuasive potential of narratives: the undecided Original Poster eventually declares to have been persuaded to consent to their child’s vaccination by one particular pro-vaccination narrative about cervical cancer. The narrative in question, however, was described by the person who posted it as difficult to share.

Inspired by this description of the persuading narrative, we explore all 120 narratives posted on the thread prior to the Original Poster’s announcement of a decision. We show how producing pro-vaccination narratives about HPV poses challenges in terms of an aspect of narratives known as ‘tellability’ – what makes the events in a narrative reportable in a particular context (Labov, 1972; Ryan, 2005). More specifically, we show how, (a) telling stories of successful vaccine uptake involves a challenge at the boundary between the non-tellable (or mundane) and the tellable and (b) telling stories about sexual activity or HPV-related illness, and particularly cervical cancer, involves a challenge at the boundary between the tellable and the untellable (or taboo). Our analysis has implications for the context-dependent nature of tellability, the role of parenting forums in vaccination-related discussions and narrative-based communication about vaccinations more generally.

## Background

The HPV virus is typically transmitted through sexual contact and can cause several conditions, including genital warts and cervical cancer. HPV vaccination is central to the World Health Organization’s strategy to eliminate cervical cancer, and has been introduced in over 100 countries (Falcaro et al., 2021). In the UK, a vaccination programme involving the bivalent Cervarix vaccine, protecting against two HPV strains, was launched in 2008 for girls aged 12–13. In 2012, Cervarix was replaced with the quadrivalent vaccine Gardasil, protecting against four HPV strains, and in 2018 the vaccination programme was extended to boys aged 12–13. Vaccination against HPV has been found to reduce the rates of cervical cancer by up to 87% (Falcaro et al., 2021). However, in 2018, vaccine coverage within target populations was estimated at 12.2% globally and at 69% among high-income countries (Spayne and Hesketh, 2021). This has been attributed to multiple factors, including safety concerns and reports of vaccine harms (Larson, 2020). The Gardasil vaccine in particular has been the focus of intense controversy and anti-vaccination sentiment around the world, due in part to the promotional strategies used by manufacturer Merck and to allegations of serious vaccine harms (Larson, 2020). Another factor is the perceived link between HPV infection and sexual activity. Parents may delay vaccination if they see it as not yet relevant for their own children, or if they fear that it will encourage them to become sexually active (Hendry et al., 2013).

Narratives have been argued to have a greater persuasive potential than non-narrative persuasive strategies (e.g. the provision of statistical information) due to the fact that cognitive and emotional involvement with story-worlds and characters may reduce awareness of persuasive intent and limit resistance to persuasion (e.g. Bilandzic and Buselle, 2012). In the context of vaccine hesitancy, Cawkwell and Oshinsky (2016) suggest that the provision of evidence from clinical findings about vaccines is no match as a persuasive strategy for highly emotional narratives of vaccine harms, and they therefore make the case for paediatricians to use narratives to address parents’ concerns about childhood vaccinations.

The experimental literature on narrative persuasion provides some support for Cawkwell and Oshinsky’s suggestion. In a study involving men who have sex with men, De Wit et al. (2008) found that risk perceptions associated with the hepatitis B virus and intentions to be vaccinated against it were highest among participants who received evidence in narrative form, rather than statistical evidence, assertions of increased risk, and no information about risk. Nan et al. (2015) compared the influence of evidence type (narrative, statistical or a hybrid of both) on unvaccinated college students’ perceptions of the risks associated with HPV infection, and found that combining both types of evidence led to the greatest increase in reported risk perceptions. First-person narratives about HPV infection and abnormal cervical smear tests were found to lead to greater risk perceptions about HPV than third-person narratives (Nan et al., 2015). And narratives of surviving cervical cancer were found to have greater persuasive potential in relation to the HPV vaccine than narratives of death caused by cervical cancer, especially when the surviving protagonist had not taken up the vaccine due to ‘social barriers’, such as the perceived association between HPV and sexual promiscuity (Krakow et al., 2017).

While experimental studies on narrative persuasion can manipulate stimulus texts to control for a range of variables, much less is known about naturally-occurring vaccination narratives, particularly in dynamic and organically evolving communicative contexts where storytelling and responses to storytelling are affected by multiple pressures and tensions, such as relevance to previous contributions, community norms, and the management of self-image and of mutual relationships. The online parenting forum Mumsnet Talk, from which our data is drawn, is one such context, and matters greatly for discussions and decisions about (childhood) vaccinations. Between 50% and 85% of people in a wide range of countries report turning to the internet for information about health-related issues (Wang et al., 2021). Parenting sites such as Mumsnet are well-documented sources of information, support and discussion about a variety of issues, including health concerns and childhood vaccinations (Betti et al., 2021). A survey by Campbell et al. (2017) reported that, out of 626 parents in England who searched the internet for information about vaccinations, 29% specifically accessed Mumsnet. As we discuss below, views and experiences about vaccinations are often shared in the form of narratives, which need to be tellable in the context in which they occur.

## Tellability

The notion of tellability or reportability, originates from sociolinguistic research on oral storytelling, where narrators need to prevent their audience from responding with ‘So what?’ or any reasonable equivalent – the clearest and most embarrassing indication of a storytelling failure (Labov, 1972). The concept has also been applied more generally in narratology to theorise the notion of narrative beyond structural properties, and to characterise different literary narrative traditions (Baroni, 2014; Kukkonen, 2017; Ryan, 2005).

Tellability is, first and foremost, a property of events as potential narrative material. Some events are inherently more tellable than others. For example, missing a flight after having one’s passport stolen en route to the airport is inherently more tellable than catching a flight as planned without incident. This captures a relatively context-independent aspect of tellability: unexpected or rare events are inherently more tellable than what is predictable or ordinary. Situations involving problems or conflict, including story participants believing or wanting different things (Ryan, 2005), are also more tellable than situations that do not involve such conflict. Particular themes have been proposed as highly tellable across cultures, such as danger and death (Ryan, 2005). However, context, broadly conceived, influences what is appropriate and relevant enough to be tellable. For example, the fact that a child agreed to take up the HPV vaccine on the condition that they would not have to have another vaccine (as in one of our examples below) is tellable on our Mumsnet thread but much less so in a speech given at a wedding.

The *way* in which a story is told also makes a difference to how tellable a narrative is perceived to be. Skilled storytellers can weave an engaging narrative out of unpromising material, while even the most reportable events may fall flat when narrated by an awkward storyteller. In the sociolinguistics tradition, the term ‘evaluation’ is used to capture the different linguistic devices that can be used to emphasise the point of a story, including ‘external evaluation’ via explicit statements such as ‘But it was quite an experience’ or ‘internal evaluation’ via intensifiers such as ‘all’ in ‘I knocked him all out in the street’ (Labov, 1972: 371, 379). More broadly, Ryan (2005) has proposed a ‘Principle of diversification’ as a way to enhance or account for the tellability of narratives. This is to do with creating semantic complexity by diversifying the ‘possible worlds’ that make up a narrative universe, for example by including failed plans, broken promises, mistaken beliefs, or other types of unrealised possibilities.

When applied to events, tellability is best seen as a cline, with a context-dependent threshold between events that are too predictable and mundane (or non-tellable) versus those that make good narrative material. While this lower-end threshold between the non-tellable and the tellable has received most attention in studies of tellability, Norrick (2005) has drawn attention to the upper-end threshold on the cline, beyond which events may be too personal, intimate or taboo to be tellable in most contexts:

The details of illness and medical procedures, sexual behaviour and fantasies etc. have no place in stories told in polite conversation for many people in most linguistic communities (Norrick, 2005: 324).

Topics that have been described as potentially untellable in previous discourse analytic studies include illness (Pearce et al., 2020), dying (Rattner, 2019), embarrassing sexual behaviours (Jackl, 2018), and some experiences associated with being a ‘bad’ parent (Jackl and McLaren, 2022; Jaworska, 2018).

Previous studies have also shown how the boundaries at both ends of the cline of tellability are not just dependent on context (from individual subjective preferences to culture), but also negotiable in context (e.g. Rajah et al., 2023). In computer-mediated communication in particular, the upper threshold of tellability may be influenced by the perception of community bonds with others and by the ‘disinhibition effect’ (Suler, 2004) that results from the ability to remain anonymous. This makes it possible to tell stories that may be too private, uncomfortable or upsetting in other contexts (Georgakopoulou, 2015). For example, Yeo (2021) shows how tellability is negotiated and co-constructed in contributions to a ‘School Secrets’ Facebook group aimed at secondary school students in Hong Kong. The option of complete anonymity makes it possible for contributors to tell normally untellable stories about mental distress and its consequences, including self-harm and suicidal thoughts.

More broadly, online discourse communities such as the contributors to particular Mumsnet threads can be seen as complex self-organising systems. These develop over time as a result of the dynamic interactions of internal and external factors operating along multiple timescales (Demjén, 2018; Semino and Demjén, 2017). From this perspective, the ‘context’ in which judgements about tellability are made is neither given a priori nor static, but rather evolves over time in an emergent fashion as posts are added by contributors (Georgakopoulou and Bolander, 2022).

In the rest of this paper, we present our data and describe how we identified and classified narratives in our analysis. After providing an overview of narratives in the data, we focus on the main pro-vaccination storytelling patterns and point out the tellability challenges they pose and how these are addressed and negotiated by contributors to the Mumsnet thread. We conclude with the implications of our findings.

## Data: A thread from Mumsnet Talk

Mumsnet was founded in 2000 with the aim to ‘make the lives of parents easier by providing them with easily accessible childcare information, advice, and solutions’ (Mumsnet, 2023a). It currently reports over 8 million user posts and 1.2 billion page-views per year, and 8 million unique visitors per month (Mumsnet, 2023b). Its users are mainly white, university educated women of childbearing age (Mumsnet, 2009), and engagement mostly takes place on the community forum section of the site called Mumsnet Talk. At the time of writing, Mumsnet Talk hosted 243 topics, including, for example, *General Health* and *Children’s Health*. Mumsnet Talk is associated with an open, straight-talking approach to parenting discussions (Pedersen and Smithson, 2013; Taylor, 2015), including on the topic of vaccinations (Coltman-Patel et al., 2022). Despite, or perhaps *because* of the perceived frank and forthright nature of this platform, contributors to particular topics and threads on Mumsnet Talk also form online discourse communities (Swales, 1990; Zappavigna, 2011) in which it is possible to share otherwise untellable parenting experiences, such as post-natal depression (Jaworska, 2018) and maternal regret (Matley, 2020).

We searched a previously created 31-million-word corpus of Mumsnet discussions of vaccinations (Coltman-Patel et al., 2022) for Original Posts (OPS) that included ‘hpv’ or ‘human papillomavirus’.^1^ This generated 130 OPs, 25 of which were found to involve indecision about whether to go ahead with an imminent HPV vaccination or delay/refuse it. Five of these threads were additionally found to include a later contribution from the Original Poster announcing that they had made a decision based on replies they had received (Semino et al., under review). As mentioned, in this paper we consider one of these threads, where the Original Poster later declares that they have come to a decision (in favour of vaccinating) as a result of a particular narrative of illness. This narrative was described by its author as difficult to share.

**Figure 1.**
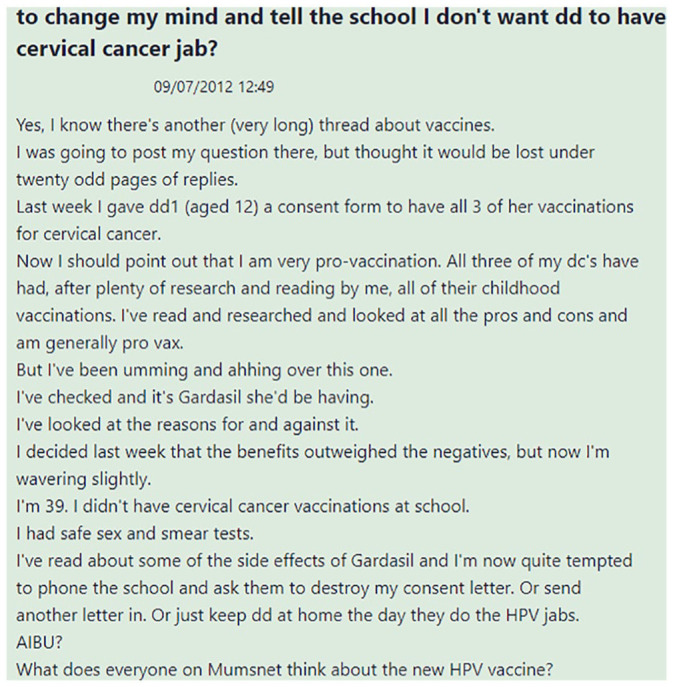
Undecided Original Post.

The thread appeared under the largest topics on Mumsnet Talk *AIBU* (*Am I being unreasonable?*) and dates from 9th July 2012, shortly after the Gardasil vaccine was approved for use in the UK. The author of the Original Post (Figure 1) describes themselves as ‘very pro-vaccination’ but reports second thoughts about having consented to their daughter receiving three doses of the ‘cervical cancer jab’ at school due to concerns about the ‘side effects’ of the newly introduced Gardasil vaccine. The writer also points out that they did not receive the vaccine themselves but had ‘safe sex and smear tests’, suggesting that this is an alternative way of keeping oneself safe from HPV infection. This leads them to consider withdrawing their consent, and to ask for advice on Mumsnet Talk.

**Figure 2.**
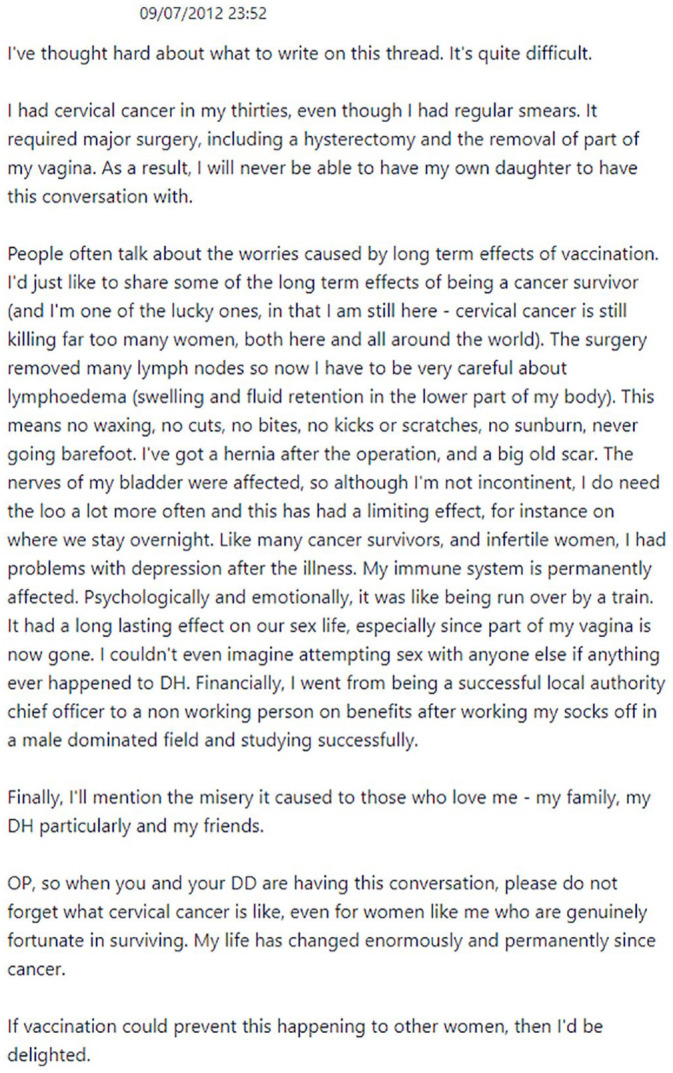
Taylor’s narrative of illness.

About 11 hours later on the same day, another contributor, who will be referred to as Taylor, posted the reply reproduced in Figure 2. After stating that they ‘thought long and hard about what to write on this thread’ as it is ‘quite difficult’, Taylor tells a lengthy personal narrative of having developed cancer that, as we explain in more detail below, includes details that would be untellable in many other contexts.

**Figure 3.**
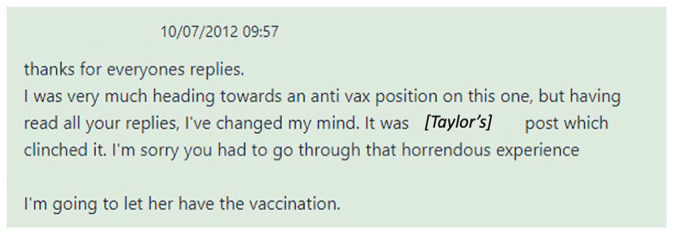
Decision announcement from the Original Poster.

This post receives several supportive and empathetic replies, for example: ‘thank you for sharing your story’ and ‘sorry for the pain you have suffered, if any post should change anti-vac views it will be yours’. The Original Poster, who had previously twice returned to the thread to add more detail about their situation, intervenes again the following morning to say that, in spite of previously ‘heading towards an anti vax position’, they have decided to consent to the vaccination thanks to the replies on the thread, and that it was Taylor’s post ‘which clinched it’ (Figure 3).

In the rest of this paper we consider this narrative in the context of the narratives in the whole thread prior to the decision announcement, in order to answer the following questions: What kinds of personal narratives are told by contributors to the Mumsnet thread? How can the notion of tellability be used to describe the challenges involved in telling different kinds of pro-vaccination personal narratives on the thread?

## Methods: Identifying and classifying narratives

At the core of most definitions of verbal narratives is the telling of a series of interconnected actions or events involving human or human-like participants that has a point (i.e. is tellable) in the context where it occurs. There is some debate, however, as to the precise criteria for classifying a stretch of text as a narrative (De Fina and Georgakopoulou, 2012: 1–25). The approach we adopted was particularly influenced by Abbott (2002), Labov (1972) and Georgakopoulou (2007).

From a narratological perspective, Abbott (2002: 24) argues that the telling of a single action or event (e.g. ‘I fell down’) qualifies as a (minimal) narrative, as it enables the receiver to infer what happened before (the person was standing up) and after (the person was on the ground). This is relevant to our data as several accounts of personal experience of HPV vaccination involve minimal reports of events such as ‘On vaccination my older ds *[dear son]* has had it’.

From a sociolinguistic perspective, Labov’s (1972) classic framework for the analysis of oral narratives involves the following five elements or stages:

- Abstract: an indication that the speaker has a story to tell and/or a brief summary.
- Orientation: a description of the setting, including time, place, people, situation.
- Complicating action: a series of clauses that present the events that are the core of the story.
- Evaluation: devices that indicate the point of the story, that is, why it is worth telling.
- Resolution: an indication of the final event.
- Coda: an indication that the story is finished and potentially some general observations on the effects of the event on the narrator.

Within this framework, only a complicating action consisting of two temporally ordered clauses is necessary for a stretch of text to be considered a narrative (e.g. ‘I tripped and fell’). The category of Evaluation is particularly relevant to tellability as it captures the linguistic devices that signal the point of a story.

More recently, sociolinguistic research on informal oral storytelling has introduced the term ‘small stories’ to capture ‘a whole range of under-represented narrative activities ranging from literally small and fragmented tellings to refusals to tell and deferrals of telling’ (Georgakopoulou, 2007; see also Bamberg, 2004 and, for applications to online storytelling, Georgakopoulou, 2015). Most relevant to our data are the following potential characteristics of small stories:

• Non- or multi-linear unfolding events sequenced in further narrative-making, not linear sequencing of past events.
• Emphasis on world-making, that is, telling of mundane, ordinary, everyday events, not world-disruption and narration of complications. (Georgakopoulou, 2015: 260).

Against this background, we analysed all 207 replies to the above OP up to the announcement of the decision for the presence and characteristics of narratives. We operationalised ‘narrative’ as the telling of one or more actions or events involving personal experiences of vaccination, HPV infection, HPV-related health concerns and illness, and other related topics. In our data, narratives are typically told in the first person and involve the author of the post and/or a family member or a friend. Each narrative was further coded for the following aspects of variation:

• Complexity, that is, whether they involved a single action/event (cf. Abbott, 2002) or two or more actions/events (cf. Labov, 1972);
• Main plot focus (e.g. vaccine uptake, illness, etc.);
• Vaccine stance (pro-vaccination, hesitant, anti-vaccination or unclear).

For all types of coding, one co-author coded the entire dataset and another co-author coded a 20% sample of the data based on a shared codebook, to establish reliability. After discussion, agreement between the two coders on the presence of a personal narrative in a post was 100%. Agreement for the characteristics of narratives was 97% for Complexity and 88.1% for both plot focus and vaccine stance, which was deemed satisfactory. Table 1 provides two examples of narrative and the relevant codes (NB: All examples are reproduced with original spelling and graphological choices.).

**Table 1.** Example narratives with coding.

Narrative	Codes
*My daughter has had the vaccine. I’d rather her be safe(r) than sorry.*	• Complexity: Single-action
	• Plot focus: Vaccine uptake
	• Vaccine stance: Pro-vaccination
*I practiced safe sex, had all the usual tests before going on the pill with ex partners and them the same. I still contracted the HPV virus and I’ve had abnormal smear tests for the past 5 years. Your daughter could only have one partner her whole life but if that partner is carrying HPV then at some point she’s going to become at risk to it. If you can reduce that risk then imo it’s worth her being vaccinated.*	• Complexity: Multi-action
	• Plot focus: Illness
	• Vaccine stance: Pro-vaccination

## Findings: Narratives on the Mumsnet thread

Overall, 120 narratives were identified in the 207 replies to the OP that precede the announcement of the decision. Other than narratives, the replies contain information relevant to the HPV vaccine (e.g. statistics about amount of protection) and a variety of other material, such as questions and expressions of opinions, e.g.: ‘How can cervical cancer be caught from boys?’ and ‘YABU’ (You are Being Unreasonable).

As shown in Table 2, the majority of narratives (*n* = 99, 82.5%) involved two or more actions/events, and the largest proportion (*n* = 77, 64.2%) had a pro-vaccination stance, with only a small number of anti-vaccination narratives (*n* = 3, 2.5%).

**Table 2.** Overview of types of narratives in Mumsnet thread.

Aspect of variation	Values	Type of narrative/total narratives (% total narratives)
Complexity	Single-action	21 (17.5)
	Multi-action	99 (82.5)
Vaccine stance	Pro-vaccination	77 (64.2)
	Hesitant	21 (17.5)
	Anti-vaccination	3 (2.5)
	Unclear	19 (15.8)
Plot focus	Vaccine uptake	41 (34.2)
	Illness	33 (27.5)
	Sex	12 (10)
	Vaccine delay/refusal	11(9.2)
	Vaccine side effects/harms	4 (3.3)
	Other	19 (15.8)

Table 2 also provides figures for plot focus groups. The two least frequent groups – Vaccine delay/refusal and vaccine side effects – were associated with a hesitant or anti-vaccination stance. Vaccine delay/refusal narratives focused on the decision-making process involving parents and daughters, and tended to link the timing of vaccination, or need for it, to the daughters’ sexual activities:

1. My DD hasn’t had it, we talked about the pros and cons and she decided she didn’t want to have it. I have said that if she even begins to think about becoming sexually active, she will need to have the jab, but (hopefully) that won’t be for at least another couple of years.

Vaccine side effects/harms narratives were relatively infrequent (*n* = 4, 3.3%), but showed how, as pointed out by Cawkwell and Oshinsky (2016), experiences of alleged vaccine harms tend to provide tellable narrative material:

2. I stated I couldn’t prove a link, but having had a very bad reaction to a vaccine 20 years ago that has forced me to spend over eight of the last 20 years fighting illhealth I think we need to know the facts. I only made a real link with the vaccine when I discovered other people who were I’ll as a result too.

In contrast, the narratives in the most frequent plot focus groups – Vaccine Uptake, Illness and Sex – were overwhelmingly pro-vaccination and focused on experiences that were more challenging in terms of tellability, either because they were relatively uneventful (Vaccine Uptake) or because they involved intimate and potentially taboo details (Sex narratives and Illness narratives). In the rest of this paper we demonstrate these challenges in tellability in pro-vaccination narratives, and show how the narratives told as the thread develops create a space in which Taylor ultimately feels able to narrate experiences that would be untellable in most other contexts.

### Pro-vaccination narratives and the lower threshold of the tellability scale: Vaccine uptake

Prior to announcing their decision, the Original Poster returns to the thread and makes the following comment:

the problem is that, as with all vaccine damage cases, millions are vaccinated, a tiny minority have complications (which could also be coincidental and not vaccine related) and it’s only ever the ones with bad experiences we hear about.

The 120 replies that precede the decision announcement do include 41 Vaccine Uptake narratives, 38 of which are pro-vaccination. Of these, however, 12 (31.6%) take the form of minimal single-action narratives (examples 3 and 4), sometimes including an explanatory or evaluative comment (example 4):

3. On vaccination my older ds [*dear son*] has had it.
4. My daughter has had the vaccine. I’d rather her be safe(r) than sorry.

Example (3) occurs at the end of a post providing advice about access to medical care, while example (4) occurs at the beginning of a post questioning ‘safe sex’ as an alternative to HPV vaccination. As Abbott (2002) points out, in these cases, readers would infer that, like the Original Poster, the respondents were invited to consent to their children being vaccinated and that they did so. The fact that no further detail is provided in each case suggests that nothing worth reporting, such as side effects or harms, happened after the vaccination.

In context, these statements function as implicit testimonies to each poster’s pro-vaccination stance and potentially as reassurance and encouragement for the undecided Original Poster to go ahead with vaccinating their child. However, a scenario in which a vaccine is administered without incident, while maximally desirable from a public health as well as personal perspective, provides little by way of tellable narrative material. Therefore, such reports of uneventful vaccine uptake lie at the lower threshold of the cline of tellability between the not-tellable and the tellable. They are relevant in context, but are minimal in length and plot, and therefore offer few opportunities for engagement with the story world and its inhabitants.

In the remaining 26 out of 38 pro-vaccination Vaccine Uptake narratives (68.4%), the challenge to tellability posed by uneventful vaccination is overcome by drawing more promising narrative material from events surrounding the vaccination, whether presented as facts or unrealised possibilities:

5. DD1 [*dear daughter 1*] had hers, after a bit of plea bargaining, she hates needles. We agreed she’d have her HPV and I’d let her off her flu one. She’s on the list for ridiculously mild asthma and reacts really badly to them (gets a really painful arm for a week, HPV didn’t bother her at all).
6. My DD *[dear daughter]* and I discussed it at length and she decided to get it done. She had been off sick with appendicitis when all the others had it. She decided to get it done and made doctors appointment herself to have it.

Example (5) mentions the daughter’s attitude to needles as a potential barrier to vaccination and includes an unrealised possibility that could have made the vaccination more eventful than it turned out to be (‘reacts really badly to them’). Several linguistic choices emphasise the point of the story by providing the writer’s evaluation, including the intensifiers ‘ridiculously’, ‘really’ and ‘at all’. Example (6) involves a different barrier to vaccination – an illness that caused the narrator’s daughter to miss out on routine vaccination at school and required her to make a separate appointment. In contrast to (5) the daughter is cast as the heroine who manages to be vaccinated against the odds, thanks to her own initiative. She is twice the grammatical subject of the verb ‘decide’, and evaluative devices emphasise her involvement in the discussion (‘at length’) and her pro-active approach in arranging the vaccination (making the appointment ‘herself’).

While the authors of these pro-vaccination narratives have to negotiate the lower threshold of the tellability cline, by contrast, contributors who tell pro-vaccination stories involving sex or illness need to negotiate the upper threshold, between the tellable and the untellable.

### Pro-vaccination narratives and the upper threshold of the tellability scale: Illness and sex

Our Mumsnet thread includes 33 personal narratives in which the writer or someone close to them experiences HPV infection, abnormal smear test results and/or cervical cancer. Of these, 20 are pro-vaccination, as in the extracts below:

7. I didn’t have sex until I was 22, never smoked, was a veggie health freak, two boyfriends, regular smears. And hey presto by 30 I had what they call ‘carcinoma in situ’ and had two big chunks of my cervix cut out. Luckily I was able to have two kids afterwards but ended up with a hysterectomy at 35 as the remaining cells were starting to change.
8. I practiced safe sex, had all the usual tests before going on the pill with ex partners and them the same. I still contracted the HPV virus and I’ve had abnormal smear tests for the past 5 years. Your daughter could only have one partner her whole life but if that partner is carrying HPV then at some point she’s going to become at risk to it. If you can reduce that risk then imo it’s worth her being vaccinated.

Generally speaking, experiences of problems and surprises are more tellable than smooth and predictable happenings. Indeed, in both examples (7) and (8) unexpected HPV-related illness provides compelling and potentially involving narrative material: repeated tests, different stages of illness, and the consequences of the illness for the authors of the post.

The particular nature of HPV infection and the context of the thread also lead to the provision of highly personal details about the contributors’ sex lives. As mentioned, the human papillomavirus is primarily transmitted through sexual contact. Changes to the cervix caused by the virus can potentially be detected via smear tests before the development of cervical cancer. Indeed, the author of the Original Post makes a reference to her own ‘safe sex and smear tests’ as potential alternatives to the vaccine for avoiding HPV infection or its consequences. In this context, as shown in examples (7) and (8), the authors of Illness narratives tend to choose to reveal their own safe and/or restrained sexual practices to suggest that these are not sufficient to protect against HPV infection and its potential consequences. In both cases, a contrast between safe sexual behaviour and the contracting of HPV or cancer is linguistically marked as part of the evaluative component of each narrative: ‘hey presto’ in the second sentence of (7) and ‘still’ in the second sentence of (8). Thus, maximising the tellability of one’s experiences of HPV-related illness in the context of the thread leads to the sharing of details that are not normally or easily tellable in other contexts, as they could be perceived as crossing the upper threshold of the tellability cline, between the tellable and the untellable.

Similar considerations apply to the 12 narratives in our data where sexual activities are the main plot focus, 10 of which are pro-vaccination. The author of extract (9) below, for example, begins by saying that the Original Poster would make ‘the wrong decision’ by withdrawing consent to their daughter’s vaccination, and then goes on to add:

9. I believe in no sex before marriage and my husband and I were both virgins when we married and we’ll be teaching our children (although I just have a boy at the moment) to believe the same, BUT that doesn’t mean they will or that that their future partners will.

Here the narrative spans both past and future events and involves both existing and hypothetical individuals to make the point that practising and teaching sexual restraint does not guarantee that one’s children will not need the protection provided by the HPV vaccine. As part of this, the writer includes the highly private detail that she and her husband did not have sex until after marriage.

While there is no linear progression in terms of plot focus or other characteristics of the narratives told by contributors, it is nonetheless notable that the ‘clincher’ narrative in Figure 2 appears as post 191 on the thread, after eight pro-vaccination Sex narratives and 18 pro-vaccination Illness narratives, three of which are more than 100 words long and occur after post 81. Taylor’s opening comments about having ‘thought hard’ about what to write because it is ‘difficult’ reflect a negotiation of the boundary between the tellable and the untellable, and prepare readers for a high degree of personal exposure. What follows is the lengthiest and most detailed narrative on the thread (315 words).

From a structural perspective, Taylor’s post includes an abstract (second paragraph), an element of orientation (‘even though I had regular smears’), a complicating action consisting of a series of events (third and fourth paragraphs), and a coda (final two paragraphs) spelling out the implications of the experience for the writer (‘My life has changed enormously and permanently’) and for the OP and other hesitant readers (‘If vaccination could prevent this happening to other women . . .’). A range of evaluative devices are spread throughout the post, including: intensifying expressions, for example, ‘major’, ‘long term’, ‘enormously’, ‘permanently’; the reference to cervical cancer causing many deaths worldwide, and to the writer being ‘one of the lucky ones’ and ‘genuinely fortunate’ in spite of everything; and the use of simile and metaphor to emphasise the consequences of illness and surgery (‘like being run over by a train’) and the professional achievements undermined by the cancer (‘after working my socks off’). The narrative also provides the perspectives of other people affected by the writer’s illness and subsequent changes (her husband, family and friends), and outlines the kinds of unrealised possibilities or virtual narratives that Ryan (2005) associates with increased tellability: the daughter that the writer will never have and the career progression she has missed out on.

However, several of the details provided in the narrative would arguably be untellable for most people in most other contexts. The writer discloses bladder problems due to nerve damage, and problems in her sex life due to the removal of part of her vagina. One of the unrealisable possibilities that are outlined in the post is finding another sexual partner if anything happened to the writer’s husband. In addition, as with Sex narratives and other Illness narratives, there is a moral angle to the identities that the writer constructs for herself. She spells out that she developed cancer in spite of keeping up with her smear tests, and emphasises how her cancer has caused a shift from a position of high social worth associated with a hard-earned professional role to being ‘a non working person on benefits’, which is implicitly presented as less worthy and potentially shameful, and therefore a challenge to her self-esteem.

In contrast with Vaccine Uptake narratives, therefore, telling pro-vaccination Illness narratives and Sex narratives in our data involves a challenge at the upper threshold of the cline of tellability, between the tellable and the untellable. This challenge is caused by the sexual mode of transmission of the virus, the fact that HPV infection and its consequences mainly (although not solely) affect the female reproductive organs and, in the Mumsnet thread under discussion, the reference to practising ‘safe’ sex in the OP. The telling of these potentially untellable elements of the narrative is facilitated not only by the anonymity provided by Mumsnet, but also by similar elements being included (with less detail and spread across narratives) in preceding posts. These collaboratively raise the upper threshold of the tellability cline for this specific thread, creating a space in which Taylor’s story can be told, albeit after an explicit negotiation of that upper threshold. As noted earlier, in this particular case, the resulting details and personal exposure are explicitly appreciated by several other contributors, and ‘clinch’ the Original Poster’s stated decision in favour of consenting to vaccination.

## Conclusions

Previous research has provided both theoretical accounts of and some empirical evidence for the persuasive potential of narratives, including in the context of vaccine hesitancy. This persuasive power has been proven particularly for HPV-related narratives of disease that are told in the first person, involve social barriers to vaccination, focus on protagonists who survive their illness, and incorporate the provision of information (Betsch et al., 2011; Krakow et al., 2017; Nan et al., 2015). However, in naturally-occurring interactional contexts, including interactions between healthcare professionals and patients, experiences relevant to HPV vaccination may not always be easy to tell. Our analysis of a Mumsnet thread where indecision about the HPV vaccine is allegedly resolved by a hard-to-tell narrative has highlighted the tellability challenges involved in sharing different types of pro-vaccination narratives about HPV.

At the lower threshold of the cline of tellability (Norrick, 2005), the challenge is caused by the limited tellability of smooth experiences of vaccination. From a public health perspective, vaccination with no or minimal side effects is the outcome that should be experienced by as many people as possible, but, inevitably, that outcome also creates poor storytelling material. The contributors to our thread mostly negotiate this tellability challenge by including additional, more tellable, details, such as difficulties around the decision-making process or unrealised situations. This – particularly acknowledging how difficult such decisions can be – is one way in which public health bodies and healthcare professionals could make uneventful vaccination stories more engaging and relatable. Moreover, although we have shown this challenge in the context of HPV vaccination, it applies to narratives of uneventful vaccine uptake more generally, including, for example against Covid-19.

Focussing on other end of the tellability cline, experimental studies showing the persuasive potential of narratives tend to involve experiences of HPV-related illness (e.g. Krakow et al., 2017) and these also form a good proportion of the narrative responses on our thread. However, we have shown that drawing such narratives from authentic personal experience can involve details about sex lives and female reproductive organs that can be uncomfortable to tell, as, depending on the context, they may approach or cross the upper threshold of the cline of tellability.

In some ways, the context of the specific Mumsnet thread we have analysed increases such challenge due to the reference to ‘safe sex’ in the OP, so that contributors offering pro-vaccination Illness and Sex narratives often mention their own sexual restraint to establish their credentials as tellers of pro-vaccination cautionary tales of HPV-related illness. This arguably makes it more likely that the undecided Original Poster will want to identify their daughter with the protagonist, but also potentially promotes problematic and highly gendered standards of sexual restraint for women in particular (Baker, 2008), and may imply that there are more or less ‘(un)deserving’ victims of HPV infection and cervical cancer (Barrera-Clavijo et al., 2016; Casper and Carpenter, 2008; Hilpert et al., 2011).

In other ways, however, the context of our specific thread also facilitates the telling of the ‘clincher’ narrative. Although there is no clear sequential progression along the cline of tellability in the narratives posted on the thread, it is nevertheless the case that the clincher narrative is preceded by several shorter Illness narratives that each contain some sensitive and personal elements. This likely makes it easier for the clincher narrative to be told (although Taylor does still provide a disclaimer) and highlights the context dependence of (un)tellability. It also points to the importance of anonymous online communities in collectively raising the upper threshold of tellability within a dynamically developing context, and enabling intimate and uncomfortable revelations to be made (Yeo, 2021). This leads to potential benefits that may outweigh the challenges we have just mentioned. Norrick (2005) suggests that stories involving self-disclosure and transgressive or taboo topics may be told in pursuit of intimacy and be appreciated by receivers because of the courage and openness demonstrated by the teller. The fact that a hard-to-tell narrative is named as the catalyst that changed the Original Poster’s mind from indecision to vaccine uptake, along with the other supportive responses it receives, are examples of this.

In public health messaging about the HPV vaccine the upper threshold of the tellability scale is not as easily negotiable, and the authenticity that is associated with narratives told on online forums such as Mumsnet Talk is hard to replicate. These issues may exacerbate the difficulties with addressing vaccine hesitancy around the only vaccine to date that has been proven to prevent a type of cancer. However, our results do point towards the crucial importance of engagement with vaccine hesitant individuals and communities over longer periods of time (to allow for trust and relationships to develop, and for stories to be told), as well as to the vital role of real lived-experience accounts and peer-to-peer interactions in addressing vaccine hesitant attitudes in ways that are appropriate for different communities at different points in time.

## Notes

1. The ethical issues involved in analysing online forum interactions are complex (Mackenzie, 2017). In Mumsnet’s case, posts are in the public domain and contributors can use pseudonyms as usernames. We did not therefore seek individual consent for our study. However, as Mumsnet owns all material posted on the site, we sought and obtained their permission to carry out the research. We also removed original usernames and any identifying information. The study was approved by the FASS-LUMS Research Ethics Committee at Lancaster University.
